# Ru@UiO-66-NH_2_ MOFs-Based Dual Emission Ratiometric Fluorescence for Sensitive Sensing of Arginine

**DOI:** 10.3390/bios14100512

**Published:** 2024-10-21

**Authors:** Jiawen Fan, Junjie Qi, Jingkun Li, Fuwei Pi

**Affiliations:** 1 State Key Laboratory of Food Science and Technology, School of Food Science and Technology, Jiangnan University, Wuxi 214122, China; 2 Collaborative Innovation Center of Food Safety and Quality Control in Jiangsu Province, Jiangnan University, Wuxi 214122, China

**Keywords:** metal–organic framework, arginine, ratiometric fluorescent, Ru@UiO-66-NH_2_

## Abstract

Arginine has been widely applied in the food industry as coloring agents, flavoring agents, and nutritional fortifiers. It is also one of the major components of feed additives. Currently, methods for the highly selective detection of arginine remain absent. For accurate and sensitive detection of L−arginine, a novel ratiometric fluorescence assay based on Ru@UiO-66-NH_2_ was developed and demonstrated in this study. Under optimized detection conditions, the limit of detection (LOD) of this assay for L-arginine was 2.32 μM, which is superior to most assays reported to date. Meanwhile, Ru@UiO-66-NH_2_ showed good stability within 30 days, demonstrating the wide applicability of the proposed assay. The spike-and-recovery rates of the proposed assay for L-arginine in real samples (e.g., tea, grape juice, and serum) were 84.27–113.09%. Overall, the proposed assay showed high sensitivity, good reproducibility, and excellent stability in the detection of L-arginine in both buffer and real samples.

## 1. Introduction

Being the elementary units of proteins, amino acids are essential for human metabolism and life activities [1,2]. Deficiency of essential amino acids causes various diseases, if not death. Arginine is an amino acid widely observed in living organisms. It is a key player in human metabolism, particularly the biological regulation of cardiovascular, immune, and endocrine systems [3,4]. Meanwhile, arginine promotes human health by increasing the number of phagocytes, facilitating wound healing, regulating vascular tension and endocrine functions, and promoting hormone secretion [5,6]. Arginine is also a crucial substance for T cell vitality in the human body, as it promotes cellular oxidative metabolism and increases the activity, persistence, and in vivo antitumor response of the cells [7]. Additionally, arginine is among the most common tumor markers, as it possibly provides nutrients for some nutrition-deficient tumors [8]. Furthermore, arginine can reduce blood glucose, impede fatty acid generation, and prevent diabetes incidence [9,10,11,12].

To date, various methods have been reported for detecting arginine, such as high-performance liquid chromatography (HPLC) [13], tandem mass spectrometry (MS/MS) [14], spectrophotometry [15], colorimetric approaches [16], and electrochemical approaches [17]. Nevertheless, these methods have different limitations. HPLC- and MS/MS-based assays are expensive and tedious and require large instruments and sophisticated sample preparation. Thus, they are unsuitable for practical applications. Spectrophotometric and colorimetric methods are limited by low specificity. Electrochemical methods require sophisticated modification of arginine-methylated proteins. Arginine detection by using specific enzymes has recently been explored. For instance, in an enzyme-based assay for detecting arginine, Madi et al. used arginine, urease, and glutamate dehydrogenase [18]. Despite improved specificity, enzyme-based methods exhibited poor performances in detecting the target in real samples because of interferences by similar substances (e.g., urea and ammonia). Therefore, an accurate, efficient, and sensitive method must be urgently developed for the real-time monitoring of arginine in food additives.

In virtue of a high sensitivity and facile process, fluorescence probes have been extensively used in bio-sensing [19,20,21]. Nevertheless, most fluorescence sensors reported so far are based on single signals, which results in poor interference resistance [22,23]. Hence, ratiometric fluorescence sensors with dual-emission characteristics have been fabricated by integrating two fluorescence signals. Such sensors have two independent fluorescence emission peaks, with one peak serving as the reference peak that remains constant and the other serving as the signal peak that may be quenched or enhanced by the target analyte. Meanwhile, the two transmission signals can be simultaneously tuned, and self-calibration can be achieved, leading to improved interference resistance and detection accuracy [24]. Among various materials, metal–organic framework (MOF) comprising metal atoms and organic linkers have received remarkable attention as a novel candidate for ratiometric fluorescence sensors [25,26] because of their tunable pore structure for loading different luminescent objects [27,28]. It is well known that the zirconium framework UiO-66 is one of the most classic, high water-stable, and luminescent MOFs. Several materials have been developed for the detection of amino acids based on the post-synthetic modification (PSM) of UiO-66. For instance, Dong et al. [29] proposed a UiO-66-NH_2_-based method for detecting arginine with a LOD of 21.50 μM. Peng et al. [30] prepared the Ag/Eu/CDs@UiO-66 composite through carbon encapsulation and developed a ratiometric fluorescence probe for arginine detection, and the LOD was 0.74 μM. By developing a ratiometric fluorescence probe via in-situ encapsulation of rhodamine B in the UiO-66-NH_2_, Chi et al. [31] demonstrated that the probe can be used for detecting arginine in serum. Zhong et al. [32] introduced Eu^3+^ ions into the MOF structure to generate a highly efficient luminescent Eu^3+^@UiO-66-FDC material for sensing tryptophan. Nevertheless, current assays are limited by low sensitivity and poor interference resistance, and therefore are unsuitable for practical applications.

**Scheme 1:**
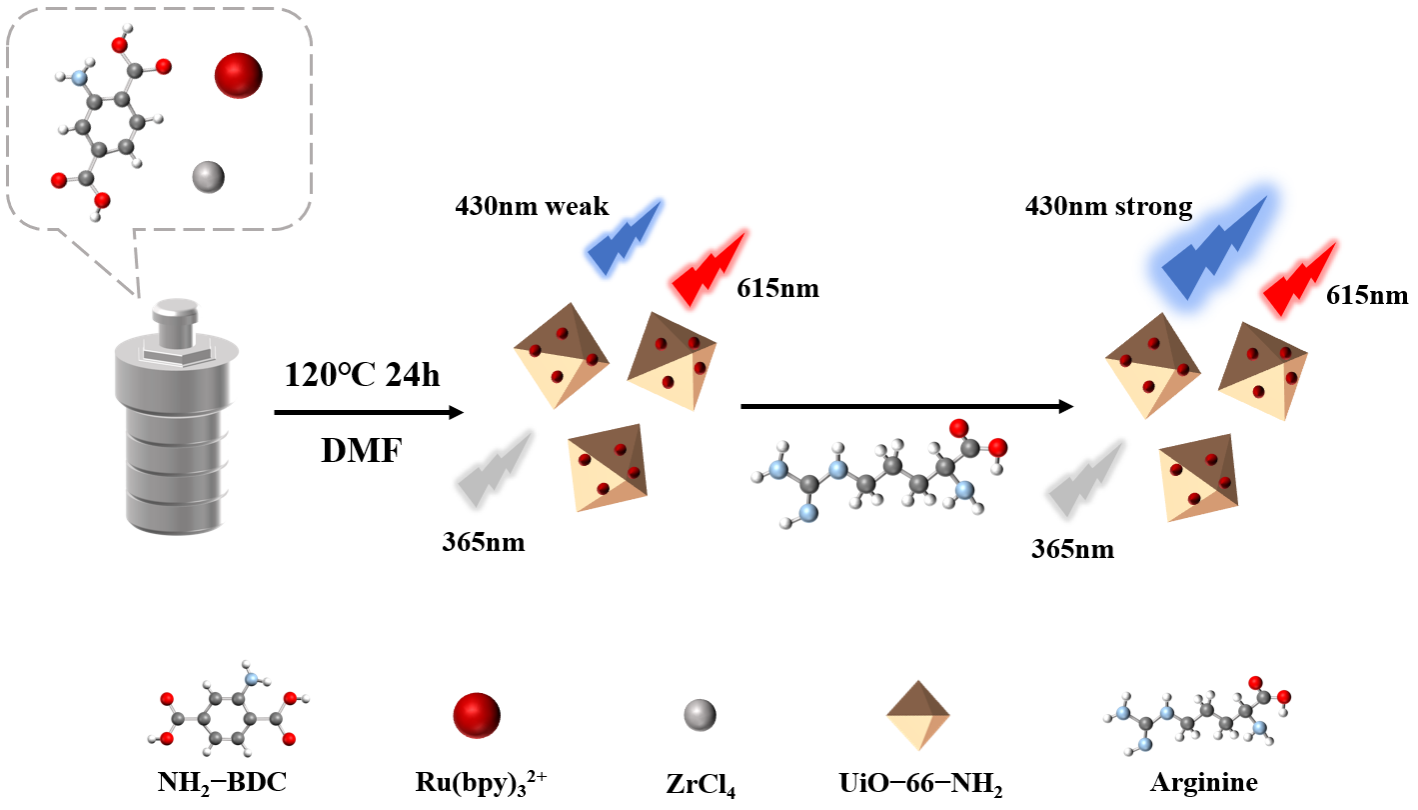
Schematic illustration of the preparation of Ru@UiO-66-NH_2_ and the ratiometric fluorescence detection of arginine.

In this study, a novel Ru@UiO-66-NH_2_-based ratiometric fluorescence probe was developed. Specifically, using ZrCl4, 2-aminoterephthalic acid (NH_2_-BDC) and Ru(bpy)_3_^2+^ as the precursors, we prepared Ru@UiO-66-NH_2_ through the one-pot solvothermal method. The developed probe was accurate for detecting L-arginine in both buffer and real samples. As shown in Scheme 1, the response of the sensor to the target is mainly reflected by changes in the fluorescence signal of zirconium-based MOF while the intensity of embedded Ru(bpy)_3_^2+^ remained constant due to its high chemical stability and photostability. Therefore, the fluorescence signal of Ru(bpy)_3_^2+^ could be used as an internal reference to promote the precision of sensing. Under optimized detection conditions, the proposed assay displayed good linearity in the 0.5 × 10^−3^–2 × 10^−3^ M range. The LOD of the proposed assay for L-arginine was 2.32 μM, which is superior to that of most assays reported to date. Ru@UiO-66-NH_2_ exhibited good stability at pH = 3–10 within 30 days, which confirmed the wide applicability of the proposed assay. Additionally, the spike-and-recovery rates of the proposed assay conducted using real samples (e.g., tea, grape juice, and serum) were 84.27–113.09%.

## 2. Materials and Methods

### 2.1. Chemicals

Tris(2,2′-bipyridine) dichlororuthenlum(II)hexahydrate (Ru(bpy)_3_^2+^) was purchased from Inno-Chem Co., Ltd. (Beijing, China). Zirconium(IV) chloride (ZrCl_4_) was purchased from Adamas Reagents Co., Ltd. (Shanghai, China). 2-Aminoterephthalic acid (NH_2_-BDC) was purchased from Shanghai Aladdin Reagent Co., Ltd. (Shanghai, China). Methanol, N, N-dimethylformamide (DMF), L-arginine (Arg), L-cysteine (Cys), L-alanine (Ala), L-leucine (Leu), L-isoleucine (Ile), L-threonine (Thr), L-tryptophan (Trp), L-phenylalanine (Phe), L-serine (Ser), glycine (Gly), L-histidine (His), L-tyrosine (Tyr), L-methionine (Met), L-glutamine (Gln), L-proline (Pro), L-valine (Val), and L-asparagine (Asn) were purchased from Sinopharm Chemical Reagents Co., Ltd. (Shanghai, China). All reagents were AR grade and were used without further purification. Ultrapure water employed in all experiment was obtained from a Millipore water purification system (18.2 MΩ·cm, Millipore, Burlington, MA, USA).

### 2.2. Synthesis of Ru@UiO-66-NH_2_ Probe

The probe of Ru@UiO-66-NH_2_ was prepared following a solvothermal method with slight modifications [33]. That is, 93.2 mg ZrCl4, 36.2 mg NH_2_-BDC, and 18 mg of Ru(bpy)_3_^2+^ were dissolved into 12 mL of DMF under sonication. Then the mixture was transferred to the polytetrafluoroethylene reactor and kept in a 120 °C oven for 24 h. The precipitate was collected by centrifuging under 10,000 rpm for 10 min and washed with DMF and methanol three times, followed by drying in vacuum at 60 °C for 10 h. As a control group, UiO-66-NH_2_ was prepared by the same protocol with no Ru(bpy)_3_^2+^ added.

### 2.3. Instruments and Data Collection

Scanning electron microscopy (SEM) images were obtained in a high-resolution scanning electron microscope (SUS8100, Hitachi, Tokyo, Japan) at an acceleration voltage of 15 kV. Transmission electron microscopy (TEM) images were obtained using a field-emission transmission electron microscope (JEOL JEM-F200, Tokyo, Japan) at an acceleration voltage of 200 kV. UV-Vis spectra were recorded on a UV-Vis spectrophotometer (T9, Beijing Purkinje General Instrument Co., Ltd., Beijing, China). X-ray diffraction (XRD) patterns were measured on a powder X-ray diffractometer (PXRD) (D2 PHASER, Karlsruhe, Germany) at scanning range = 5°–50°, step = 0.05°, scanning rate = 0.5°/min equipped with the Cu-k-α (30 kV and 10 mA) as the source. Fourier-transform infrared spectroscopy (FT-IR) and fluorescence spectra were recorded on a spectroscopy spectrophotometer (IS10, Nicolet, Waltham, MA, USA) and fluorescence spectrometer (Fluoro Max4, Horiba JY, Irvine, CA, USA), respectively.

### 2.4. Fluorescence Detection of L-Arginine

Fluorescence evaluation of L-arginine with the Ru@UiO-66-NH_2_ probe was operated as follows: first, 50 mg of Ru@UiO-66-NH_2_ powder was dissolved in 5 mL of water with sonication for 30 min. Then series of L-arginine standard solutions with concentrations from 5 × 10^−3^ to 2 × 10^−2^ M were prepared. Finally, 40 μL of the L-arginine standard solution, 40 μL of Ru@UiO-66-NH_2_ solution, and 320 μL of aqueous solution with pH = 7 were mixed and incubated at 25 °C for 4 min for the fluorescence measurements. Parameter settings of fluorescence spectrometer were as follows: excitation wavelength of 365 nm, scanning speed at 2000 nm/min.

The calibration curve of the fluorescence intensity ratio (F_430_/F_615_) and concentration of L-arginine could be developed, and the LOD can be determined by the following:

LOD = 3σ/k(1)

where k is the slope of the calibration curve and σ is the standard deviation of 15 blank signals. Each experiment was performed in three replicates.

### 2.5. Recovery Rate Measurement

Food samples of tea and grape juice were purchased from the local market. Mice were purchased from Beijing Vital River Laboratory Animal Technology Co., Ltd. (Beijing, China). The serum collected from mice was first centrifuged under 8000 rpm for 15 min, the supernatant was diluted to 100-fold through adding ultrapure water. Subsequently, three L-arginine standard solutions with pre-decided concentrations were added to these samples. After incubation for 4 min, the fluorescence intensities of the probes were recorded, and the recovery rates were determined by the following:

Recovery rate (%) = C_1_/C_0_ × 100% (2)

where C_1_ is the calculated concentration of L-arginine based on the calibration curve and C_0_ is the spiked concentration of L-arginine.

## 3. Results and Discussion

### 3.1. Characterization of Ru@UiO-66-NH_2_ Probe

**Figure 1:**
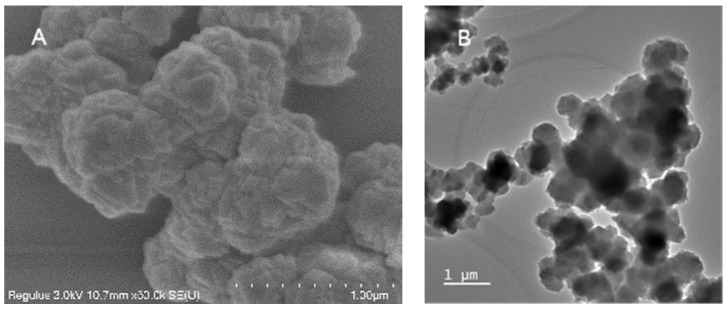
The (**A**) SEM and (**B**) TEM images of Ru@UiO-66-NH_2_.

Figure 1A,B display the SEM and TEM images of the obtained Ru@UiO-66-NH_2_, respectively. According to the images, it can be easily observed that the fabricated Ru@UiO-66-NH_2_ probe is irregularly shaped and had sizes of 200–400 nm. Compared with UiO-66-NH_2_ [34], Ru@UiO-66-NH_2_ probe exhibits smoother edges and corners, but holds an extended outermost shell due to the revulsive crystallization of the Ru^2+^ ion.

**Figure 2:**
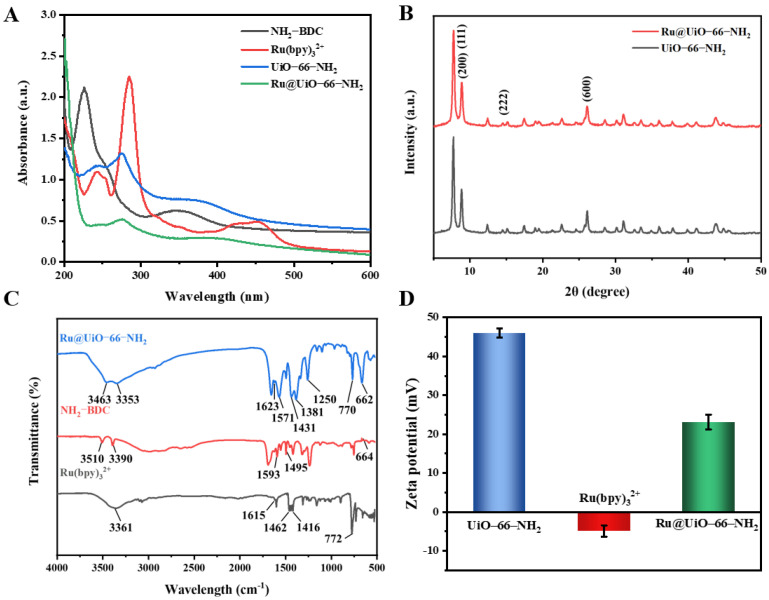
(**A**) UV–Vis spectra of NH_2_-BDC, Ru(bpy)_3_^2+^, UiO-66-NH_2_, and Ru@UiO-66-NH_2_; (**B**) XRD patterns of Ru@UiO-66-NH_2_ and UiO-66-NH_2_; (**C**) FT-IR spectra of Ru@UiO-66-NH_2_, NH_2_-BDC, and Ru(bpy)_3_^2+^; (**D**) Zeta potentials of UiO-66-NH_2_, Ru(bpy)_3_^2+^, and Ru@UiO-66-NH_2_.

To reveal the probe features, UV-Vis spectroscopy and XRD and FT-IR spectroscopy were measured as plotted in Figure 2. As shown in Figure 2A, the ligand NH_2_-BDC exhibits distinct absorptions at 246 nm and 349 nm. After self-assembly with metal-oxo clusters, UiO-66-NH_2_ showed two main absorption peaks around 275 nm and 369 nm. Notably, a broad band presents at 369 nm that was assigned to the π→π* electronic transition of aromatic rings [35]. Ru(bpy)_3_^2+^ presents two characteristic peaks at 285 nm and around 450 nm. In contrast to the spectrum of UiO-66-NH_2_, the intensity of Ru@UiO-66-NH_2_ has a sharp decrease. Additionally, for the Ru@UiO-66-NH_2_ composite, the absorption of UiO-66-NH_2_ and Ru(bpy)_3_^2+^ appears simultaneously, suggesting the successful preparation of the probe.

Figure 2B displays the recorded XRD patterns of Ru@UiO-66-NH_2_ and UiO-66-NH_2_. The characteristic peaks at 7.7°, 8.9°, 14.5°, and 26° correspond respectively to (111), (200), (222), and (600) crystal planes of UiO-66-NH_2_, and the two patterns exhibited similar peaks, indicating that the crystal structure was not damaged during the in situ preparation, which is consistent with previous studies [33,36].

Figure 2C shows the FT-IR spectra of Ru@UiO-66-NH_2_, NH_2_-BDC, and Ru(bpy)_3_^2+^. The Ru@UiO-66-NH_2_ composite displays some characteristic peaks that belong to UiO-66-NH_2_. Two typical peaks at 662 and 770 cm^−1^ are derived from the asymmetric vibration of the Zr-O bond. The peak at 1381 cm^−1^ is indicative of Zr-O-H group vibration, and the peaks at 1431 cm^−1^ and 1571 cm^−1^ are attributed to the C-O-Zr group vibration. The peaks at 3463 cm^−1^ and 3353 cm^−1^ corresponding to symmetric and asymmetric stretching vibrations of the –NH_2_ bond indicate that the functional groups in the ligand are preserved during the construction of Ru@UiO-66-NH_2_. Additionally, Ru@UiO-66-NH_2_ shows characteristic peaks of Ru-derived pyridine groups at 1250 cm^−1^ and 1623 cm^−1^, demonstrating the encapsulation of Ru(bpy)_3_^2+^ into UiO-66-NH_2_.

Figure 2D shows that UiO-66-NH_2_ has positive charges in aqueous solutions attributed to the amino groups on the framework. Ru(bpy)_3_^2+^ is negatively charged with zeta potentials of −4.89 mV, whereas the zeta potential of Ru@UiO-66-NH_2_ increases to +23.09 mV, revealing the potential electrostatic interaction of Ru(bpy)_3_^2+^ and UiO-66-NH_2_ that promote their incorporation.

### 3.2. Optimization of Detection Conditions

**Figure 3:**
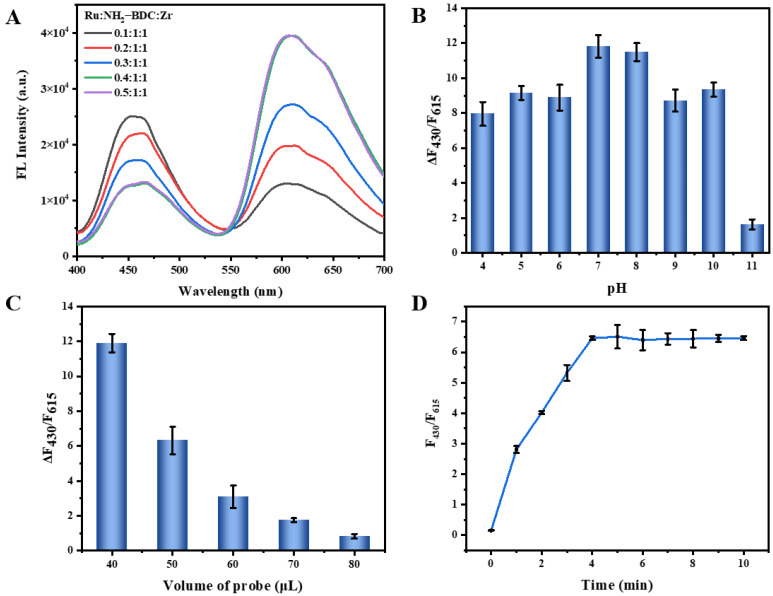
(**A**) Fluorescence intensity of Ru@UiO-66-NH_2_ at different Ru(bpy)_3_^2+^/NH_2_-BDC ratios; (**B**) Fluorescence intensity of Ru@UiO-66-NH_2_ at different pH values; (**C**) Fluorescence intensity of Ru@UiO-66-NH_2_ at different probe sizes; (**D**) Fluorescence intensity of Ru@UiO-66-NH_2_ under different reaction times.

First, the amount of Ru(bpy)_3_^2+^ was optimized. To determine the Ru/NH_2_-BDC ratio, the molar ratio of NH_2_-BDC and Zr was established to be 1:1. Complexes loading different amounts of Ru(bpy)_3_^2+^ were prepared, then the fluorescent properties were explored for determining the optimized Ru/NH_2_-BDC ratio. As plotted in Figure 3A, there was a negative correlation between the fluorescence intensity of UiO-66-NH_2_ and the load of Ru, whereas that of Ru(bpy)_3_^2+^ was positively related to the Ru(bpy)_3_^2+^/NH_2_-BDC ratio. The fluorescence intensity exhibited no further increase once the Ru/NH_2_-BDC ratio exceeded 0.4:1, since the Ru(bpy)_3_^2+^ load was maximized. Therefore, the feed ratio of Ru@UiO-66-NH_2_ was established to be 0.4:1.

Then pH was optimized. Specifically, the same amount of Ru@UiO-66-NH_2_ powder was added to ultrapure water with different pH values, with the L-arginine concentration maintained at 1.7 × 10^−2^ M. The solution was subsequently kept stationary at room temperature for 10 min, and the fluorescence intensity was recorded. A solution without L-arginine was considered the control group. The optimized pH was 7 (Figure 3B). Since most amino acids were water-soluble, Ru@UiO-66-NH_2_ was directly dissolved in ultrapure water for sensing without adjusting the pH.

Later, the Ru@UiO-66-NH_2_ probe concentration was optimized. At a constant L-arginine concentration (1.7 × 10^−2^ M), excessive Ru@UiO-66-NH_2_ reduced sensitivity. Therefore, 40 μL (i.e., 1 mg/mL) Ru@UiO-66-NH_2_ was used for detecting L-arginine (Figure 3C).

Additionally, we determined the effects of reaction times on detection performances. Specifically, the F_430_/F_615_ ratio of the detection system with L-arginine at 1.7 × 10^−2^ M was monitored within 10 min. The ratio increased drastically within 4 min, after which the ratio was saturated. Hence, 4 min was considered the optimized reaction time (Figure 3D).

### 3.3. Fluorescence Sensing on L-Arginine

**Figure 4:**
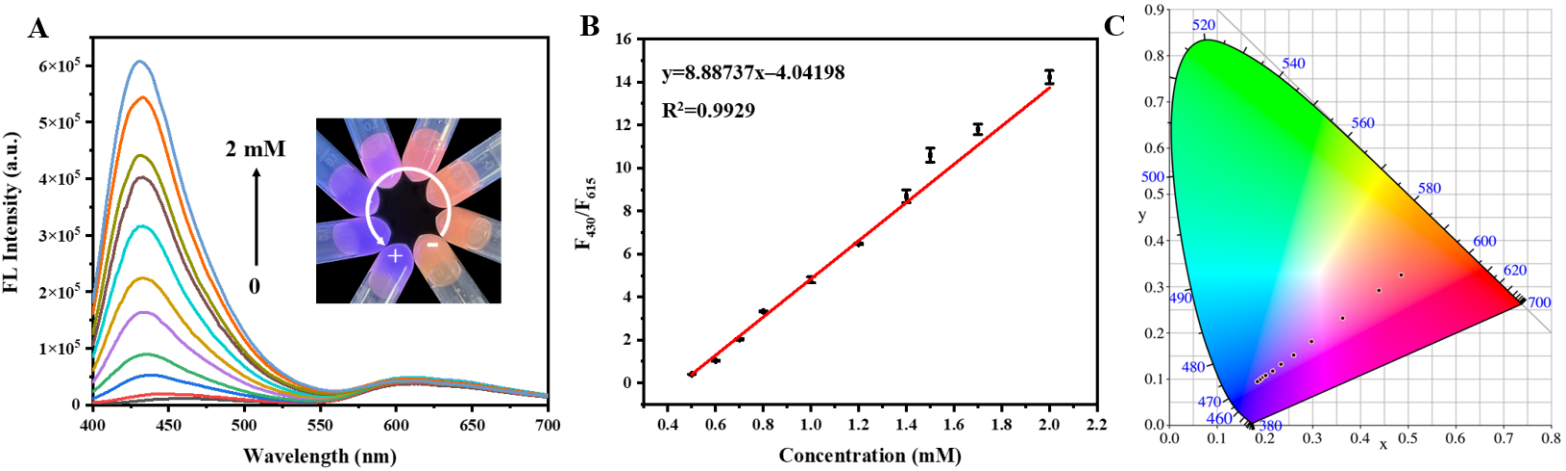
(**A**) Fluorescence emission spectra at 365 nm excitation in the presence of different L-arginine concentrations; (**B**) Linear fitting curve of fluorescence; (**C**) CIE chromaticity.

To further explore the fluorescence sensing of L-arginine by Ru@UiO-66-NH_2_, L-arginine was quantitatively detected under optimized conditions. Ru@UiO-66-NH_2_ exhibited dual-emission characteristics at a single excitation of 365 nm (Figure 4A). Emission peaks at 430 and 615 nm were attributed to the UiO-66-NH_2_ and Ru(bpy)_3_^2+^, respectively. At 0–2 mM, when the L-arginine concentration increased, the fluorescence intensity of Ru@UiO-66-NH_2_ at 430 nm increased, whereas that of Ru(bpy)_3_^2+^ at 615 nm exhibited negligible variation. Moreover, the fluorescence of Ru@UiO-66-NH_2_ at 365 nm single excitation changed from orange to blue with an increase in the L-arginine concentration, which is consistent with CIE chromaticity (Figure 4C).

The F_430_/F_615_ ratio exhibited a good linear correlation with the L-arginine concentration in the 0.5 × 10^−3^–2 × 10^−3^ M range, with a correlation coefficient (*R*^2^) of 0.9929 (Figure 4B). The LOD was 2.32 μM. Overall, the probe served as a ratiometric fluorescence sensor with good selectivity for L-arginine.

### 3.4. Selectivity and Specificity for Sensing L-Arginine, and Stability of Ru@UiO-66-NH_2_

**Figure 5:**
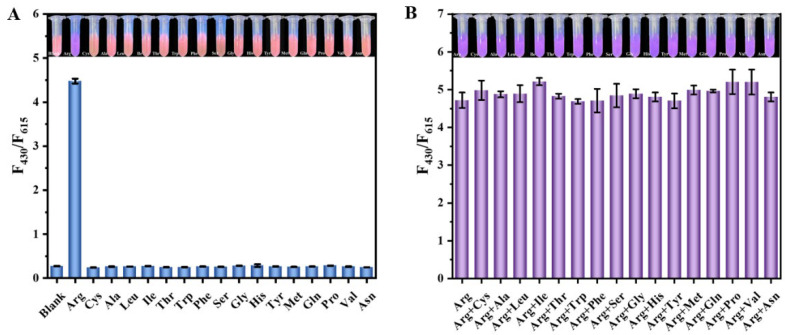
The specific FL response of different interfering substances (**A**) without and (**B**) with L-arginine.

In addition to sensitivity, selectivity is a key indicator of sensor performance in practical applications. The selectivity of Ru@UiO-66-NH_2_ for L-arginine among different amino acids was also investigated. The concentration of L-arginine was 30% of other interfering amino acids in the system for quantitative analysis. The F_430_/F_615_ ratio increased drastically upon the addition of L-arginine, but not for 16 other amino acids (Figure 5A). This demonstrated that the Ru@UiO-66-NH_2_ probe is not responsive to other amino acids. Additionally, in the presence of interfering substances, Ru@UiO-66-NH_2_ still has a stabilized response to L-arginine (Figure 5B), which demonstrated that the Ru@UiO-66-NH_2_ probe could selectively detect L-arginine in mixtures of various amino acids. Thus, the Ru@UiO-66-NH_2_ probe exhibits good selectivity and specificity that can be used for detecting L-arginine in the food matrix.

**Figure 6:**
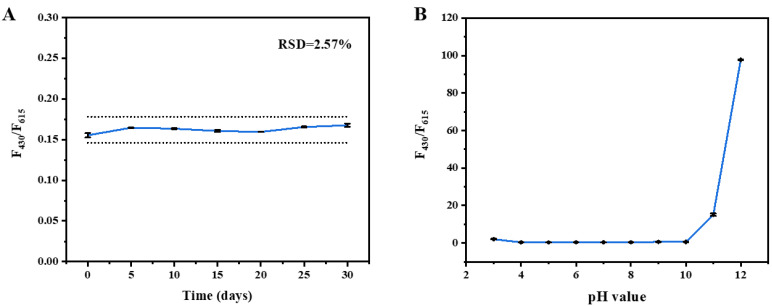
(**A**) Storage stability and (**B**) pH stability of Ru@UiO-66-NH_2_.

The storage stability and pH stability of Ru@UiO-66-NH_2_ were investigated. The F_430_/F_615_ ratio remained constant after storage for 1 month at room temperature, which indicated the good storage stability of the probe (Figure 6A). Furthermore, the ratio remained constant at a pH of 3–10 (Figure 6B), but increased gradually once pH was >11.0, which can be attributable to the partial rupture of MOF.

### 3.5. Sensing Performance in Real Samples

To clarify the sensing performance of the Ru@UiO-66-NH_2_ probe in complex systems, tea, grape juice, and serum were selected as references for real system detection. As shown in Table 1, the detection recovery rates of these groups were 84.27–113.09%, with relative standard deviations (RSDs) of <4%. Overall, the sensor is reliable as a tool for detecting L-arginine in real samples. Additionally, the performance of the as-prepared sensor and those in previous reports on L-arginine evaluations were compared (Table 2). Obviously, the Ru@UiO-66-NH_2_ probe exhibited good sensitivity and LOD.

## 4. Conclusions

In summary, a novel Ru@UiO-66-NH_2_-based ratiometric fluorescence nanoprobe was developed. Because of its intrinsic self-calibration capability, this probe demonstrated excellent anti-interference performances, thereby providing augmented sensitivity in the quantitative analysis. Under optimized detection conditions, the proposed assay for L-arginine displayed good linearity in the 0.5 × 10^−3^–2 × 10^−3^ M range. The LOD of the proposed assay was 2.32 μM, which is superior to that of most reported assays. Meanwhile, Ru@UiO-66-NH_2_ demonstrated good stability at pH = 3–10 within 30 days, which showcased the wide applicability of the proposed assay. The spike-and-recovery rates of the proposed assay conducted using real samples (e.g., tea, grape juice, and serum) were 84.27–113.09%, with relative standard deviations of <4%. Furthermore, the proposed assay can effectively detect L-arginine in real samples. Overall, this study offers a rapid and efficient method for detecting L-arginine, as well as references for selecting and optimizing methods for the rapid and quantitative detection of other amino acids.

## Figures and Tables

**Table 1:** Analytical recoveries of L-arginine in real samples.

Samples	Added (mM)	Found (mM)	Recovery (%)	RSD (*n* = 3) (%)
	1.7	1.92	113.09	1.74
Green tea	0.9	0.79	87.59	1.69
	0.5	0.48	95.93	0.06
	1.7	1.50	87.95	1.22
Grape juice	0.9	0.76	84.27	1.10
	0.5	0.48	96.65	0.09
	1.7	1.91	112.15	3.91
Serum	0.9	0.90	99.68	0.99
	0.5	0.48	95.38	3.10

**Table 2:** Comparison with other L-arginine detection methods.

Sensors	Method	Linear Ranges	LOD	References
P-sulphonato calix capped gold nanoparticles	UV–vis	4 × 10^−6^–10^−4^ mol L^−1^	4.0 μM	[37]
Fe_3_O_4_@SiO_2_-AgNPs	UV–vis	30–60 μM	2.56 μM	[38]
NH_2_-MIL-101-Al	Fluorescence	0.2–1.0 mM	45.1 μM	[39]
Tb-MOF	Fluorescence	0–2.31 mM	7.06 μM	[40]
AgInSe_2_@ZnS	Fluorescence	0.2–1 mM	26 μM	[41]
Rhodamine-thiourea/Al^3+^ complex	Fluorescence	0–1.2 × 10^−4^ mol L^−1^	2.3 μM	[42]
Eu@ZnMOF	Fluorescence	0.05–0.5 mM	20.0 μM	[43]
CDs	Fluorescence	27–107 μM	9.16 μM	[44]
UiO-66-NH_2_	Fluorescence	0–0.645 mM	21.5 μM	[29]
Ru@UiO-66-NH_2_	Fluorescence	0.5–2 mM	2.32 μM	This work

## Data Availability

The data presented in this study are available on request from the corresponding author.
